# *Staphylococcus aureus* FadB is a dehydrogenase that mediates cholate resistance and survival under human colonic conditions

**DOI:** 10.1099/mic.0.001314

**Published:** 2023-03-22

**Authors:** Amjed Alsultan, Gemma Walton, Simon C. Andrews, Simon R. Clarke

**Affiliations:** ^1^​ School of Biological Sciences, University of Reading, Whiteknights, Reading, RG6 6EX, UK; ^2^​ Food Microbial Sciences Unit, Department of Food and Nutritional Sciences, University of Reading, Whiteknights, Reading, RG6 6AP, UK; ^†^​ Present address: Department of Internal and Preventive Medicine, College of Veterinary Medicine, University of Al-qadisiyah, Aldewanyiah, Iraq

**Keywords:** *Staphylococcus aureus*, FadB, dehydrogenase, cholate, bile acids, colon

## Abstract

*
Staphylococcus aureus* is a common colonizer of the human gut and in doing so it must be able to resist the actions of the host’s innate defences. Bile salts are a class of molecules that possess potent antibacterial activity that control growth. Bacteria that colonize and survive in that niche must be able to resist the action of bile salts, but the mechanisms by which *S. aureus* does so are poorly understood. Here we show that FadB is a bile-induced oxidoreductase which mediates bile salt resistance and when heterologously expressed in *Escherichia coli* renders them resistant. Deletion of *fadB* attenuated survival of *S. aureus* in a model of the human distal colon.

## Introduction

Infection by *Staphylococcus aureus* is a leading cause of community-acquired and nosocomial disease. Its ability to colonize the nares, which occurs in 20–25 % of the population at any one time [1, 2], is linked to infection which frequently occurs when the *S. aureus* spreads to normally sterile parts of the body such as the bloodstream [3]. While this has been well characterized, several recent studies have indicated that colonization of the intestine by *S. aureus *, which occurs in *c*. 20 % of individuals and has been much less well characterized, may have important clinical implications [4]. Carriage studies of methicillin-resistant *S. aureus* (MRSA) have reported gastrointestinal colonization in 11–89 % of those who were carrying the bacterium [5–9]. Such individuals display an increased frequency of skin colonization [10].

*S. aureus* intestinal colonization can serve as an important source of transmission when faecal contamination of the adjacent environment occurs [11–14], while screening for faecal carriage is proposed as a measure to reduce transmission [15]. A study of a long-term outbreak with *S. aureus* sequence type 228 (ST228) in a Swiss hospital reported persistence of a single clone which was adapted to colonize the rectum as the primary colonization niche, over the nares [16].

Although the extent and clinical implications of intestinal colonization by *S. aureus* are still relatively ill defined [17], it can be assumed that carriage is a risk for intestinal infection; *S. aureus* can cause pseudomembranous colitis that is histologically distinct from that caused by *Clostridiodes difficile* [18]. A study of intensive care and liver transplant units showed that patients with both rectal and nares colonization by MRSA was associated with a significantly higher risk of disease (40 %) than did patients with nasal colonization alone (18 %) [9]. Multiple studies have demonstrated frequent intestinal colonization in infants, particularly those who were breast fed, and that there is a positive correlation with the development of allergies [19–23]. While a role for *S. aureus* intestinal carriage in the development of systemic *S. aureus* disease has not been established, colonization of the intestinal lumen of mice can result in the pathogen crossing the intestinal epithelial barrier and spreading to mesenteric lymph nodes [24, 25]. Furthermore, a Trojan horse model has been proposed where intestinal colonization is a putative source of *S. aureus *-infected neutrophils which then disseminate the pathogen around the body [26].

The antibacterial activity of bile salts represents a survival challenge for bacteria in the gut [27] and helps to direct the structure of the microbiome, but some pathogens use them as an environmental signal to regulate colonization and virulence [28, 29]. Many members of the microbiota initiate bile acid metabolism via bile salt hydrolases, which hydrolyse and deconjugate the glycine or taurine from the sterol core of the primary bile acids. The deconjugated bile acids can subsequently undergo a variety of microbiota-induced transformations.

Bacteria employ a number of strategies in order to survive the antibacterial activity of bile salts. Gram-negative bacteria are generally more innately bile resistant than Gram-positive bacteria due to the presence of an outer membrane, which acts as a barrier [27] which with maintenance of membrane integrity by cell envelope lipopolysaccharide (LPS) imparts protection against the actions of bile salts [30, 31]. A number of pathogens possess bile efflux pumps, including *S. aureus* which uses MnhF to resist unconjugated bile acids and survive under conditions modelling the human colon [32]. The efflux pump AcrAB in *Salmonella enterica* serovar Typhi and *S. enterica* serovar Typhimurium allows these pathogens to grow at bile concentrations that are much higher than those encountered *in vivo* [33]. Similarly, HefC is an AcrB homologue that confers bile salt resistance to *Helicobacter pylori* [34]. The multidrug efflux pump CmeABC of *Campylobacter jejuni* mediates bile salt resistance and is required for colonization of chickens [35].

Thus bile salt resistance is important for intestinal survival of several enteric bacteria and while there is currently only limited understanding of how *S. aureus* resists bile, we have previously reported the role of MnhF in bile salt efflux [32]. Here, FadB, a putative oxidoreductase, was enriched in the cell envelope of bile-treated *S. aureus *, suggesting that it is involved in bile resistance and therefore survival of the pathogen under conditions that mimic the human colon.

## Methods

### Bacteria, plasmids and growth conditions

The bacterial strains and plasmids used in this study are listed in Tables 1 and 2, respectively. *Escherichia coli* strains were grown in lysogeny broth (LB) medium using selection with ampicillin at 100 µg ml^−1^ where appropriate. *S. aureus* was grown in Tryptic Soy Broth (TSB; Sigma), with inclusion of the following antibiotics, where appropriate: erythromycin at 5 µg ml^−1^ and lincomycin at 25 µg ml^−1^. Phage-mediated transductions were performed as described previously [36].

**Table 1.** Bacterial strains

Strain	Description/genotype	Source or reference
* S. aureus* SH1000	Wild-type	[87]
* S. aureus* RN4220	Accepts *E. coli* DNA	[88]
* S. aureus* Δ*fadB*	Δ*fadB* mutation in SH1000	This study
* E. coli* DH5α	F^–^ φ80*lac*ZΔM15 Δ(*lac*ZYA-*arg*F)U169 *rec*A1 *end*A1 *hsd*R17(r_K_^–^, m_K_^+^) *pho*A *sup*E44 λ^–^ *thi-*1 *gyr*A96 *rel*A1	Invitrogen
* E. coli* BL21 (DE3)	F^–^ *omp*T *hsd*S_B_ (r_B_^–^, m_B_^–^) *gal dcm* (DE3)	Invitrogen
* E. coli* BW25113	*Δ(araD-araB)567 ΔlacZ4787*(::rrnB-3) *λ^-^ rph-1 Δ(rhaD-rhaB)568 hsdR514*	[89]
* E. coli* JW3822	* E. coli* BW25113 *fadB*	[89]

**Table 2.** Plasmids

Plasmid name	Description	Antibiotic resistance	Source or reference
pMAD	Temperature-sensitive (30 °C) *E. coli – S. aureus* shuttle vector. pE194^ts^:: pBR322	Ap^R^ (* E. coli *) Em^R^ (* S. aureus *)	[90]
p*∆fadB*	pMAD-based vector for *∆fadB* mutation	Ap^R^ (* E. coli *) Em^R^ (* S. aureus *)	This work
pBAD His A	Expression vector containing *araBAD* promoter	Ap^R^	[91]
p*fadB*	pBAD His A containing *fadB* internal fragment	Ap^R^	This work
pET21a	His_6_ tag overexpression vector	Ap^R^	Novagen
pAmjed1	pET21a containing internal fragment encoding rFadB	Ap^R^	This work

### Preparation of cell envelope material

Cell envelope was extracted based on a previously described method [37]. Growing mid-log cultures of *S. aureus* in TSB (37 °C with orbital shaking at 250 r.p.m.) were harvested and diluted as appropriate to an optical density at 600 nm of ˜0.6 and resuspended to and OD_600_ of 0.6. Then, 50 ml was centrifuged at 16 100 ***g*** for 5 min at 4 °C, resuspended, and washed in 1 ml of TBS [50 mM Tris-HCl (pH 7.5), 0.1 M NaCl, 0.5 mM PMSF, 1 mg of iodoacetamide ml^–1^). Samples were centrifuged at 16 100 ***g*** for 5 min at 4 °C, and pellets were resuspended in 1 ml of TBS. Next, 0.5 ml of suspension was added to tube containing Lysing Matrix B (MP Biomedicals) containing glass beads, which was then shaken 10 times in a FastPrep-24 machine (MP Biomedicals) set at speed 60 for 40 s. The tubes were placed on ice and allowed to cool between each cycle. Glass beads were allowed to settle, and the supernatant containing insoluble cell wall material was removed. Insoluble material was recovered by centrifugation at 16 100 ***g*** for 10 min at 4 °C and washed in 1 ml cold 50 mM Tris-HCl (pH 7.5) followed by centrifugation at 16 100 ***g*** for 10 min at 4 °C before resuspension in SDS-PAGE buffer.

### SDS-PAGE

Proteins were separated by SDS-PAGE with a 4 % (w/v) stacking gel and a 12 % (w/v) resolving gel in a Mini-Protean II gel apparatus (Bio-Rad).

### Quantitative real-time PCR

mRNA from *S. aureus* was quantified using quantitative real-time PCR (qRT-PCR). Cells were grown as described above and then treated with RNAlater stabilization solution (Invitrogen), and RNA was isolated using RNeasy Mini Kits as per the manufacturer’s instructions. DNA was removed using Turbo DNase (Invitrogen). The quantity and quality of purified mRNA was determined using an Agilent RNA 6000 Nano Kit and Bioanalyzer. A total of 0.5 µg of RNA was reverse transcribed using the Tetro cDNA synthesis kit (Bioline) and reactions lacking RNA or reverse transcriptase were included as controls. qRT-PCR was performed using the Agilent qPCR system and iQ SYBR green supermix (Bio-Rad). Relative amounts of transcript were determined by relative quantification using *gyr* as the internal comparator gene, based on consistent levels observed in previous studies [38–41]. The oligonucleotides used for qRT-PCR are listed in Table 3.

**Table 3.** Oligonucleotides; restriction endonuclease sites are underlined

Name*	Sequence 5′−3′
FadBUpFor^1^	CTAAATGGATCCACAGTCACATGAACTGCG
FadBUpRev^2^	TTACCCGGGTTGTCATAGTGATTCCTCCAATTTAGTTG
FadBDownFor^2^	CATTACCCGGGCGTAATTAAAAGATAGTCATTAAGAGAGG
FadBDownRev^1^	CGTTTGGGATCCAGAAGCAAATGCTTCGTTCAATTCG
FadBOverFor^3^	GGAGATATACATATGATTGGAGGAATCACATATGAC
FadBOverRev^4^	GTGGTGGTGCTCGAGATTACGTAATGGCTTA
FadBCloneFor**^5^**	CTAAGAGCTCATTGGAGGAATCACTATGACAATTAATAAAG
FadBCloneRev^1^	GACTAGGTACCTCTTTTAATTACGTAATGGCTTACCAG
*fadB*For	CACGGTCTATGTCTCGGAAATC
*fadB*Rev	CAAGACGAAGCGGGACTATTT
*gyrB*For	ATCGACTTCAGAGAGAGGTTTG
*gyrB*Rev	CCGTTATCCGTTACTTTAATCCA
Sau	GAAGCAAGCTTCTCGTCCG

*Restriction sites: ^1^ *Bam*HI, ^2^ *Xma*I, ^3^ *Nde*I, ^4^ *Xho*I, ^5^ *Sac*I.

### Generation of an *fadB* mutant

To generate a Δ*fadB* mutant, DNA fragments corresponding to ~1 kb upstream and downstream of *fadB* were amplified using Pwo polymerase (Roche) with oligonucleotide pairs FadBUpFor/FadBUpRev and FadBDownFor/FadBDownRev (Table 3). PCR products were purified and then digested with *Bam*HI/*Sma*I and cloned into pMAD. The resulting plasmid was used to transform electrocompetent *S. aureus* RN4220. Plasmid was transduced into *S. aureus* SH1000 using ϕ11 phage. The temperature-sensitive nature of plasmid replication was exploited to integrate the plasmid into the bacterial chromosome, by plating cells onto medium containing erythromycin and lincomycin at 42 °C. After further rounds of plating, erythromycin- and lincomycin-sensitive colonies were isolated and the loss of *fadB* was confirmed by PCR. Use of both erythromycin and lincomycin reduces the chance of unintentional selection of point mutantions.

### Determination of MIC

The MICs of selected bile salts, sodium cholate (CA), sodium deoxycholate (DCA), sodium chenodeoxycholate (CDCA), sodium glycocholate (GCA) and sodium taurocholate (TCA) were determined by broth dilution. MICs were determined by stepwise dilutions and were reproduced in three independent experiments.

### Time course measurement of bacterial viability upon exposure to bile salts

Overnight cultures of *S. aureus* were grown to mid-exponential phase in TSB at 37 °C with shaking. After harvesting, cells were washed twice with sterile 5 mM HEPES buffer (pH 7.2) containing 10 mM glucose and then resuspended in the same buffer to an OD_600_ of 0.5. Cells were incubated with various concentrations of bile salt which give reliable kill curves, for 30 min at 37 °C. At 10 min intervals, dilutions from each of the bile salt-treated groups were made with a sterile peptone saline diluent (Oxoid). Dilutions were plated onto tryptic soy agar plates and incubated overnight at 37 °C. Colonies were counted, and percentage viabilities were calculated based on the initial untreated cell suspension.

### Cloning and overexpression of *fadB*

The *fadB* gene was amplified from *S. aureus* SH1000 DNA by PCR using Phusion DNA polymerase (Thermo Scientific). Oligonucleotides FadBOverFor and FadBOverRev were used to amplify the gene. PCR products were digested with *Nde*I and *Xho*I and ligated into similarly digested pET21a. The ligation mixture was transformed into *E. coli* DH5α, and transformants were selected for resistance to ampicillin (Ap^r^) and checked by restriction digestion and sequenced to confirm the fidelity of the PCR. A representative plasmid, pAmjed1, was transformed into *E. coli* BL21(DE3).

For overexpression in *E. coli* BW25113, oligonucleotides FADBcloneFor and FADBcloneRev were used to generate a PCR product which was subsequently digested with *Sac*I and *Kpn*I and ligated into similarly digested pBAD/HisA to create plasmid pAmjed2, where *fadB* is fused to P_BAD_, which is under the tight control of the arabinose-inducible AraC-controlled promoters [42, 43].

### Overexpression and purification of recombinant FadB

His6 tag recombinant rFadB was expressed by addition of 100 µM IPTG to growing cells. Purification was achieved using a pre-packed Ni-Sepharose column with the Biologic HR workstation (Bio-Rad). Eluted fractions were analysed by SDS-PAGE, and the protein concentration was determined using Bradford reagent. The validity of the rFadB protein overexpression was confirmed by submitting purified rFadB for MS analysis at the University of Birmingham. The protein sample was digested with trypsin and the masses of the recovered peptides were determined by LC-MS.

### Measurement of FadB enzyme activity

A kinetic spectrophotometric assay was used to measure the enzymatic activity of recombinant FadB based on a previously described method [44]. The enzyme converts acetoacetyl-CoA to β-hydroxy butyryl-CoA in the presence of β-NADH. This reaction was measured by recording the decrease in NADPH absorption at 340 nm. One unit of enzyme activity was defined as conversion of 1 µmol acetoacetyl-CoA to β-hydroxy butyryl-CoA per minute at pH 7.3 at 37 °C in the presence of NADH.

### Batch culture distal colon model system

An *in vitro* anaerobic batch culture system was used to simulate the main physiological and microbiological processes in the distal colon, including residence time, substrate availability and pH [45, 46]. The experiment was carried out in triplicate using faecal samples from three healthy volunteers. After obtaining verbal informed consent, a standard questionnaire to collect information regarding health status, drug use, clinical anamnesis and lifestyle was administered before the donor was asked to provide a faecal sample. No volunteers had received antibiotics, commercial probiotics or prebiotics, steroids or other drugs proven to have an impact on gut microbiota for at least 3 months before sampling. None of them had any history of gastrointestinal disorders. All healthy faecal donors had the experimental procedure explained to them and were provided with an opportunity to ask questions. The University of Reading research ethics committee exempted this study from review because no donors were involved in any intervention and waived the need for written consent because the samples were not collected by means of intervention. All faecal samples were collected on site, kept in an anaerobic cabinet (10 % H_2_, 10 % CO_2_, 80 % N_2_) and used within 15 min of collection. Samples were prepared on the day of the experiment and within 1 h of production, and were diluted to 1 : 10 (w/v) in anaerobic phosphate buffer (0.1 M; pH 7.4). Samples were homogenized in a stomacher for 2 min and the resulting slurry was inoculated into batch culture fermenters. The model was inoculated with *S. aureus* (˜2×10^10^ c.f.u. ml^−1^) as a single dose, suspended in colonic model media.

Survival of *S. aureus* was enumerated by fluorescence *in situ* hybridization (FISH), an efficient method for enumerating specific species in a mixed culture. Samples for FISH were fixed immediately in 4 % paraformaldehyde as described previously [47, 48], using Cy3-labelled Sau probe (Sigma-Aldrich) (Table 3), which is specific for this species [49].

## Results

### FadB is a bile salt-induced cell envelope protein

**Fig. 1.**
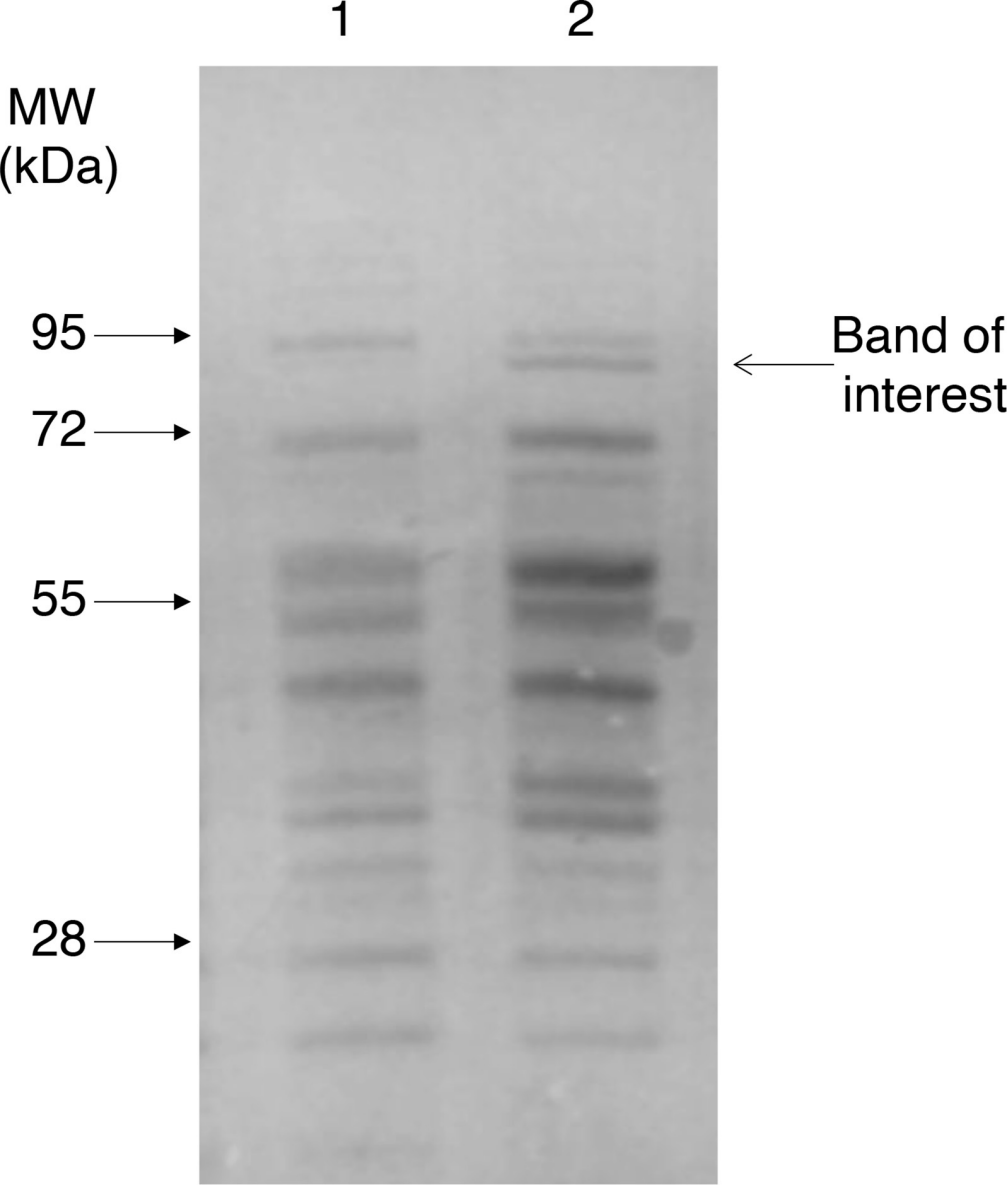
Cell envelope proteins in bile-treated *S. aureus *. Coomassie-stained SDS-PAGE (12 %, w/v) gel of cell envelope extracts grown in the absence (lane 1) or presence (lane 2) of bovine bile (8 %, w/v).

To determine whether exposure to bile salts caused differences in the cell envelope protein profile of *S. aureus* SH1000, cells were cultured in the presence of bile (8 % w/v bovine bile salts; Oxoid), which does not impede growth, until they reached OD_600_ ˜0.6 in TSB. SDS-PAGE showed the presence of a bile-induced protein of approximately 85 kDa (Fig. 1). The band was excised from the gel and submitted for analysis by MS (University of Birmingham, UK), revealing it to be a putative 3-hydroxyacyl-CoA dehydrogenase encoded by *fadB* (Table S1, available with the online version of this article).

**Fig. 2.**
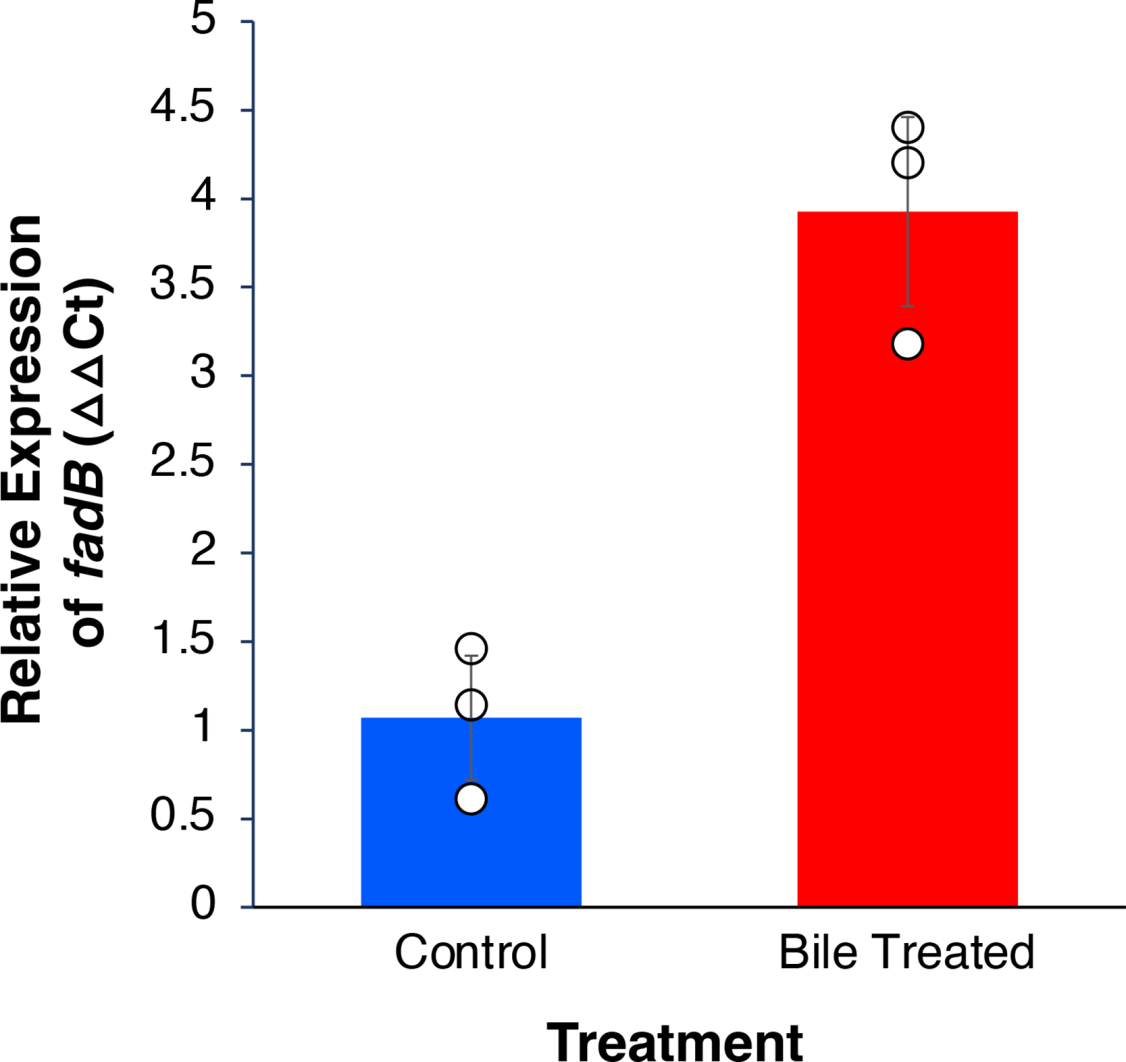
Transcription of *fadB* is upregulated in the presence of bile acids. qRT-PCR was performed to quantify amounts of transcript in *S. aureus* SH1000. Data represent means±sd from three independent experiments. White circles indicate data points. *P*<0.01, Student’s *t*-test.

Transcription of *fadB* was measured using qRT-PCR to determine whether transcription of the gene is induced by bile salts. *S. aureus* was grown in the presence of bile as described above and the levels of transcripts were quantified. The level of *fadB* mRNA in bile-treated cells was approximately four times higher than in untreated cells (Fig. 2).

### FadB mediates resistance to bile salts

**Fig. 3.**
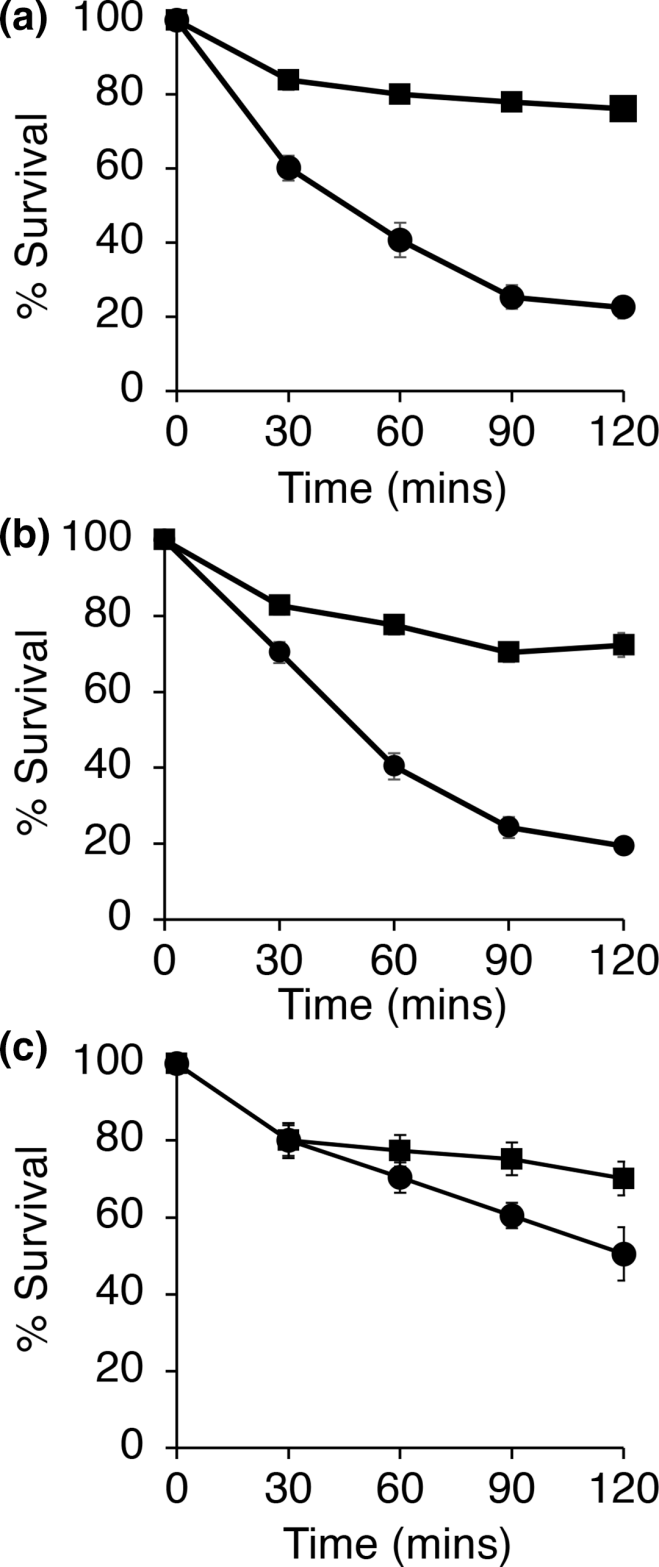
FadB protects *S. aureus* against the bactericidal activity of bile salts. Viability of *S. aureus* SH1000 (■) and Δ*fadB* (•) treated with (**a**) 2 mM cholic acid, (**b**) 0.25 mM deoxycholic acid and (**c**) 25 mM glycocholic acid. Data represent means±sd from three independent experiments. *P<0.05; all other time points *P*>0.05, Student’s *t*-test.

We hypothesized that as FadB is found in the cell envelope of bile-treated cells, it may influence *S. aureus* bile acid sensitivity. To test this, an unmarked, in-frame Δ*fadB* strain was created in *S. aureus* SH1000. A mutant lacking an antibiotic resistance phenotype was necessary for subsequent use of our colonic model, where adding such genes to complex mixtures of gut microbes should be avoided. The mutant strain had no growth defect when grown on/in TSB solid or liquid medium in the absence of bile salts (results not shown). A selection of bile acids with differeing pKa values were used to test susceptibility of the mutant. *S. aureus* Δ*fadB* had a 2–3-fold reduced MIC for cholic acid and deoxycholic acid, but not chenodeoxycholic acid or conjugated bile acids (Table 4). In killing assays, the Δ*fadB* strain was significantly more sensitive than the parent (Fig. 3). Increased sensitivity of the mutant strain was only observed with certain unconjugated bile salts but it should be noted that as in previous studies, we were unable to determine the *S. aureus* MIC of conjugated bile salts as they were insoluble at >200 mM [32].

**Table 4.** MICs of bile salts for *S. aureus* SH1000 and Δ*fadB*

Bile salt	Wild-type (mM)	Δ*fadB* (mM)
CA	22	7
DCA	1.2	0.6
CDCA	1.2	1.2
GCA	>200	>200
TCA	>200	>200

CA, sodium cholate; CDCA, sodium deoxycholate; DCA, sodium deoxycholate; GCA, sodium glycocholate; TCA, sodium taurocholate.

**Fig. 4.**
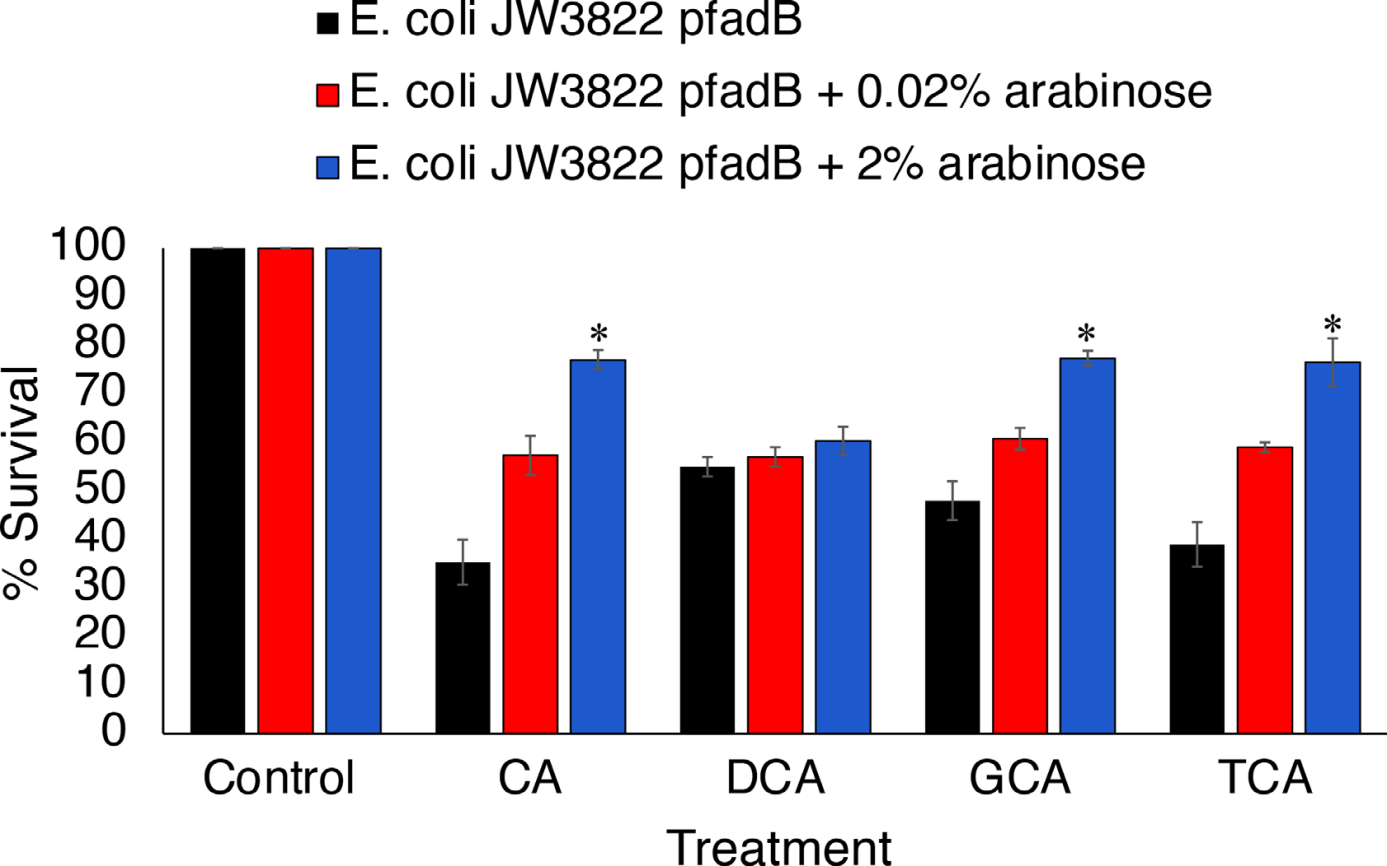
Heterologous expression of FadB in *E. coli* protects against the bacteriostatic effects of bile salts. Data show the viability of *E. coli* JW3822 (*fadB*) and *E. coli* JW3822 (p*fadB*) cells in LB medium containing cholic acid (CA, 10 mM), deoxycholic acid (DCA, 2 mM), glycocholic acid (GCA, 50 mM) and taurocholic acid (TCA, 50 mM) and then grown for 16 h at 37 °C. Cell counts were determined by viable plate counting. Data represent means±sd from three independent experiments. *P<0.005, Student’s *t*-test of arabinose treated versus no arabinose.

To confirm a role for *fadB* in resistance to bile salts, the gene was cloned under the control of the arabinose-inducible inducible P_BAD_ promoter of plasmid pBAD/HisA, which allowed arabinose dose-dependent expression of FadB in *E. coli* JW3822, an isogenic *fadB* mutant of *E. coli* BW25113. *E. coli* JW3822 had a lower MIC for cholic acid, glycocholic acid and taurocholic acid than its parent (Table 5). Expression of FadB increased the MIC of cholic acid and conjugated bile salts in an arabinose-dependent manner (Table 5) and exclusion of arabinose reduced the MIC to the same level as the background strain lacking the plasmid. Similarly, expression of FadB in *E. coli* also decreased the bacteriostatic effects of bile salts on that bacterium in an arabinose dose-dependent fashion (Fig. 4).

**Table 5.** MICs of bile salts for wild-type (BW25113), *fadB* mutant (JW3822) and recombinant *E. coli* expressing FadB at different levels of arabinose induction

Bile salt	* E. coli* BW25113	* E. coli * JW3822	* E. coli * JW3822 pBAD	* E. coli* JW3822 p*fadB*		
				0 % Arabinose	0.02 % Arabinose	2 % Arabinose
CA	60	30	30	30	50	50
DCA	4	4	4	4	4	4
CDCA	4	4	4	4	4	4
GCA	120	80	80	80	100	100
TCA	120	80	80	80	100	100

Inclusion of 2 % arabinose did not affect the MIC of the control strains.

CA, sodium cholate; CDCA, sodium deoxycholate; DCA, sodium deoxycholate; GCA, sodium glycocholate; TCA, sodium taurocholate.

### *S. aureus* FadB is a dehydrogenase

**Fig. 5.**
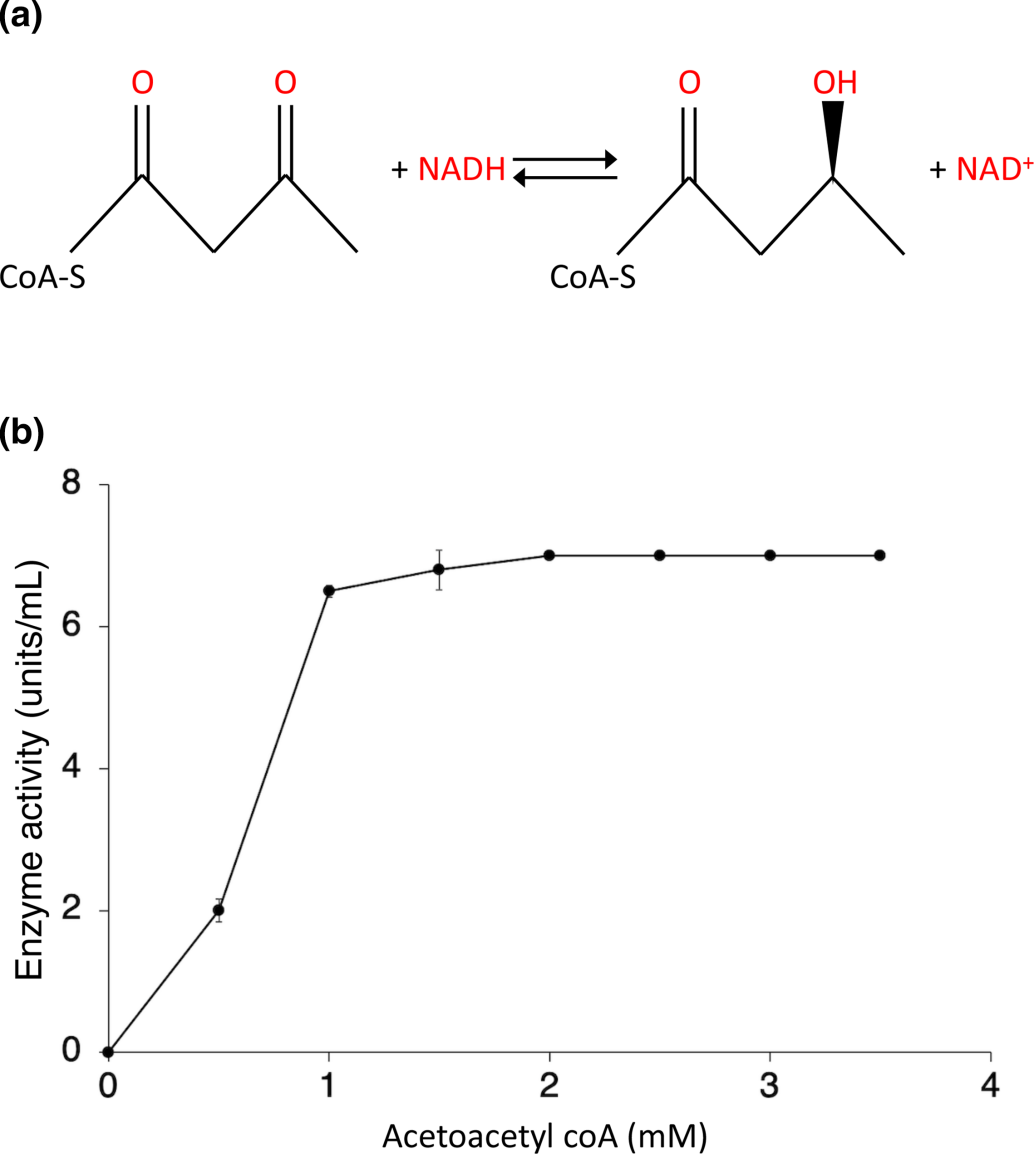
Hydroxyacyl-CoA dehydrogenase enzyme assay. (**a**) Conversion of acetoacetyl-CoA to hyrdoxybutyryl-CoA in the presence of β-NADH. (**b**) The catalytic activity of the enzyme by converting acetoacetyl-CoA to hydroxybutyryl-CoA in the presence of NADH as a cofactor was determined spectrometrically (*A*_340_). The serial dilution of the substrate (acetoacetyl-CoA) was used to measure the activity rate of the enzyme. Data represent means±sd from three independent experiments. No activity was observed in the absence of enzyme.

FadB is proposed to convert acetoacetyl-CoA to hyrdoxybutyryl-CoA in the presence of β-NADH (Fig. 5a). Using purified rFadB (Fig. S1) this activity was demonstrated by measuring the decrease of NADPH absorption at 340 nm as described previously [44]. The enzyme showed catalytic activity at 0.53.5 mM of substrate acetoacetyl-CoA in the presence of 0.1 mM NADH (Fig. 5b), but no activity was observed in the absence of rFadB. Thus, *S. aureus* FadB was demonstrated to exhibit dehydrogenase activity in the presence of acetoacetyl-CoA.

### FadB is required for survival of *S. aureus* in a human gut model

To examine the role of FadB in survival of *S. aureus* under conditions found in the human distal colon, we used a temperature- and pH-controlled faecal batch culture model system (37 °C, pH 6.8) containing bile. *In vivo* studies of colonic bacteria are hampered by a lack of suitable animal models as they do not correctly simulate the physicochemical conditions or gut microbiota found in the human colon. We have previously used similar *in vitro* models to study the survival of *S. aureus* and the impact of infection on the host’s colonic microflora [32, 48].

**Fig. 6.**
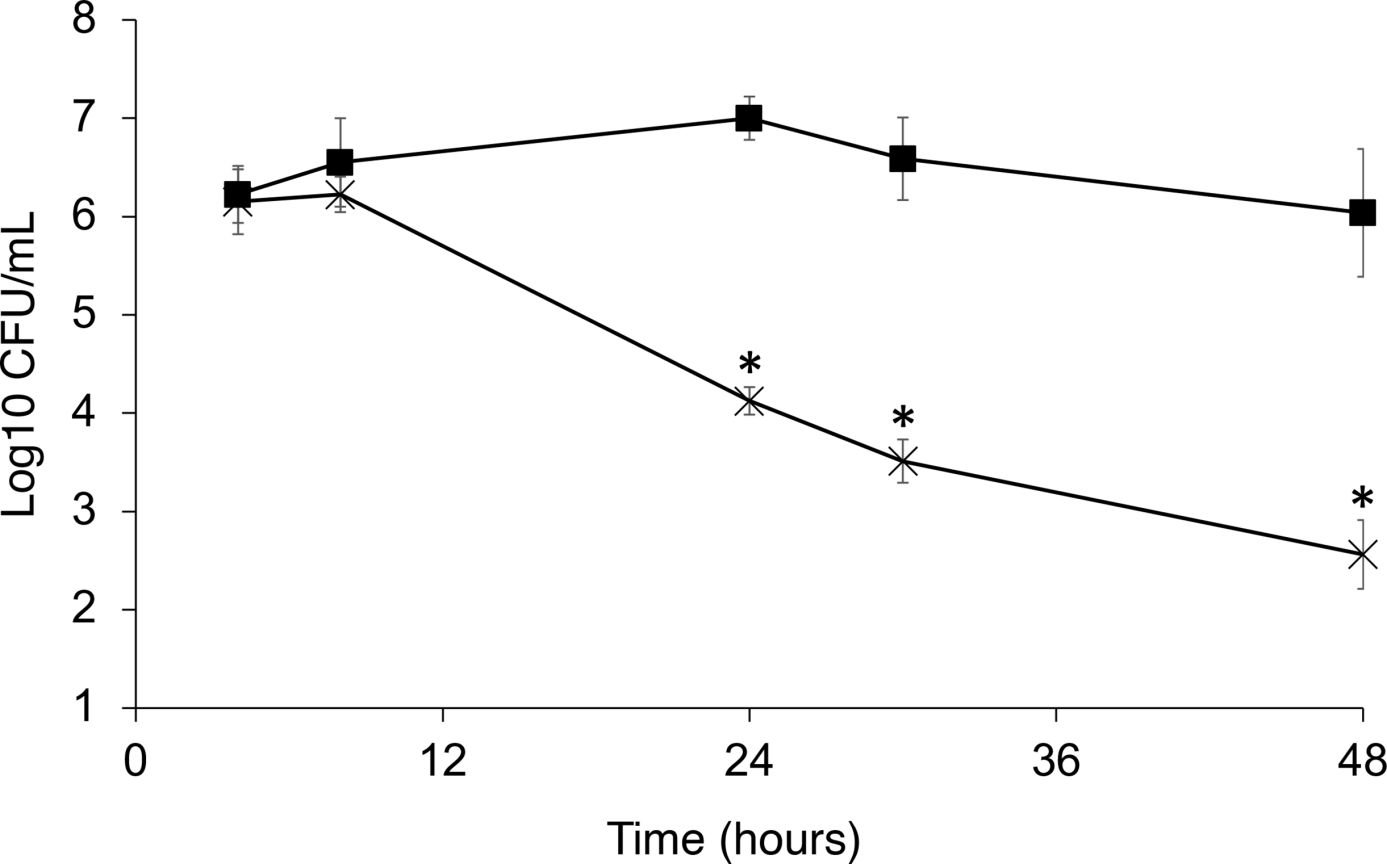
FadB is required for *S. aureus* survival in a human distal colon model. Survival of *S. aureus* SH1000 (■) and Δ*fadB* (×) cells in a human colonic model. Samples were taken at 4, 8, 24 and 48 h post-infection. Data represent means±sd from three independent experiments. *P<0.01, Student’s *t*-test.

We ran parallel models, each containing either *S. aureus* Δ*fadB* or the parental wild-type. The culture vessel was inoculated with *S. aureus* to a final concentration of 10^10^ c.f.u. ml^−1^ in a single dose. Survival of *S. aureus* Δ*fadB* was significantly attenuated compared to that of its parental strain (Fig. 6). Thus, FadB mediates *S. aureus* survival under human colonic conditions.

## Discussion

The interaction between *S. aureus* and its human host is complex and is built upon a range of interactions and adaptations. As a pathogen of great medical significance and as a common commensal, *S. aureus* must be able to resist host innate antimicrobials such as peptides, fatty acids and bile, a complex cocktail composed principally of bile salts, phospholipids, cholesterol, proteins and bilirubin [50]. The human liver secretes up to 1 litre of bile per day into the gut [27] and molecules secreted by bacteria during infection, including *S. aureus *, are an important cause of metabolic cholestasis, an inability of hepatocytes to produce bile [51]. Additionally, bile salts are present in human serum at micromolar concentrations [52–54].

In addition to anti-bacterial effects, bile salts serve endocrine functions [55–57], consequently regulating their own synthesis, conjugation, transport and detoxification, as well as lipid, glucose and energy homeostasis [58]. Moreover, bile salts have an important role in maintaining intestinal barrier function and induce genes encoding antimicrobial peptides and lectins [59]. Thus, by modulating the composition of the bile acid pool in the gut, bacteria can exert multiple effects on host physiology.

Many antibacterial agents act by disrupting the cytoplasmic membrane resulting in loss of proton gradients and electrical potential across the membrane, leading eventually to cell death [60]. Due to their structural and chemical properties, bile salts are generally considered to be weak acids which decrease intracellular pH and dissipate transmembrane potential. In *S. aureus* and other bacteria, bile salts act by disrupting the cytoplasmic membrane, which results in dissipation of the membrane’s proton gradient and electrical potential, resulting in cell death [61, 62].

In the human colon, bile salts are modified by the normal microbiota [63]. The ‘gateway’ modification is usually regarded as hydrolysis of an amino acid conjugate by bile salt hydrolase [64]. However, unconjugated bile salts are more active against *S. aureus* than either glycocholic or taurocholic acids [32, 62]. Major modifications include deconjugation, oxidation of hydroxyl groups at C-3, C-7 and C-12, and 7α/β-dehydroxylation [65, 66]. The ability of bacteria to remove the 3-, 7- and 12-hydroxyl groups is dependent in part on 3α-, 7α- and 12α-hydroxysteroid dehydrogenase (HSDH) activity [67–71]. Members of the gut microbiome are capable of removing the 7α-hydroxyl group from cholic acid and chenodeoxycholic acid, forming deoxycholic acid and lithocholic acid, respectively [66, 72]. Deoxycholic acid and chenodeoxycholic acid are the principal bile acids found in the stool of healthy humans [73].

In *E. coli *, *fadB* encodes a 3-hyrdroxyacyl-CoA dehydrogenase [74, 75] involved in fatty acid degradation [76]. Bile stress commonly induces proteins involved in fatty acid metabolism [77] and, in *Salmonella enterica *, *fad* genes are upregulated in response to bile exposure [78].

In response to the detergent action of bile acids, bacteria change the lipid metabolism and therefore the lipid and protein profiles of their cell membrane, which can lead to alterations in the physical properties of the membrane [79, 80]. Modification of fatty acid composition can maintain membrane fluidity, a phenomenon known as homeoviscous adaptation [81]. In *Lactobacillus reuteri *, these changes include decreased amounts of phospholipids and a lower ratio of saturated to unsaturated fatty acids, influencing the physical properties of the cell membrane, potentially adapting the bacterium to the conditions found in the human gut [82].

In this study, we show that FadB, a putative 3-hydroxyacyl-CoA dehydrogenase, protects *S. aureus* against the bactericidal activity of bile acids, including cholic acid. Bacteria commonly modify bile acids *in vivo* including dehydrogenation, and thus the activity of FadB may result in a derivative of cholic acid that is less toxic to *S. aureus *.

Alternatively, bile salts have well-characterized effects on bacterial membranes, showing greater lytic activity towards membranes with increasing fluidity [83]. FadB shortens fatty acids [84–86] and may thus increase membrane fluidity, rendering the cell more susceptible to the surfactant nature of bile acids. It remains to be determined whether either of these two phenomena accounts for the resistance phenotype observed in this study, but it seems entirely plausible that either or both could, at least in part, account for our observations.

## Supplementary Data

Supplementary material 1
