# Supplementary material for: Staphylococcus aureus FadB is a dehydrogenase that mediates cholate resistance and survival under human colonic conditions
Source: Microbiology (Reading). 2023 Mar 22;169(3):001314. doi: 10.1099/mic.0.001314 (PMC10191381; doi:10.1099/mic.0.001314)
Supplement: Supplementary material 1 [file mic-169-1314-s001.pdf]

Accession	Description	Protein	Score	Coverage	# Proteins	# Unique Peptides	# Peptides	# PSMs	# αα	MW (kDa)	Calc. pI
A0A0H2XFB6	3-hydroxyacyl-CoA dehydrogenase OS=Staphylococcus aureus (strain USA300) GN=SAUSA300_0226 PE=3 SV=1 - [A0A0H2XFB6_STAA3]	FadB	505.77	61.49	12	3	42	159	753	84.6	6.00
A0A0D1JV66	3-hydroxyacyl-CoA dehydrogenase OS=Staphylococcus aureus subsp. aureus GN=QU38_14655 PE=3 SV=1 - [A0A0D1JV66_STAAU]	FadB	461.17	58.57	4	1	40	147	753	84.6	6.00
A0A0U1MW86	Elongation factor G OS=Staphylococcus aureus GN=fusA PE=3 SV=1 - [A0A0U1MW86_STAAU]	FusA	161.02	54.40	21	24	24	40	693	76.5	4.88
Q2FI09	Phosphoribosylformylglycinamidine synthase subunit PurL OS=Staphylococcus aureus (strain USA300) GN=purL PE=3 SV=1 - [PURL_STAA3]	PurL	142.28	56.24	17	28	28	39	729	79.5	4.88
Q2YSD6	ATP-dependent Clp protease ATP-binding subunit ClpC OS=Staphylococcus aureus (strain bovine RF122 / ET3-1) GN=clpC PE=3 SV=1 - [CLPC_STAAB]	ClpC	137.70	41.32	13	27	29	39	818	91.0	5.73
A0A122EPB9	Formate acetyltransferase OS=Staphylococcus aureus GN=pflB PE=4 SV=1 - [A0A122EPB9_STAAU]	PlfB	136.15	42.99	7	26	26	40	749	84.8	5.48

Supplementary Table 1. Mass Spectrometry data, top 6 results. Band of interest as per Figure 1.

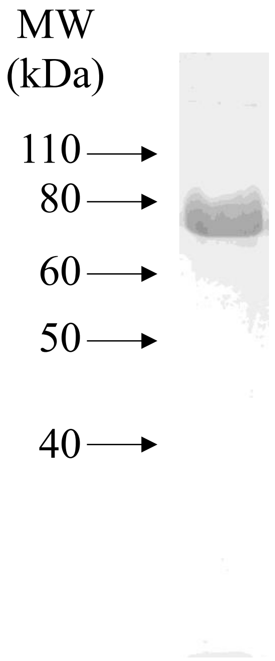

Supplementary Figure 1. Purification or rFadB. *c*. 99% pure as determined by SDS-PAGE (12% w/v).
